# Dataset for transcriptome analysis of *Salmonella enterica* subsp. *enterica* serovar Typhimurium strain 14028S response to starvation

**DOI:** 10.1016/j.dib.2020.106008

**Published:** 2020-07-07

**Authors:** Natalia E. Gogoleva, Vladimir Ya. Kataev, Alexander S. Balkin, Andrey O. Plotnikov, Elena I. Shagimardanova, Anastasia M. Subbot, Sergey V. Cherkasov, Yuri V. Gogolev

**Affiliations:** a Kazan Institute of Biochemistry and Biophysics, FRC Kazan Scientific Center of RAS, 2/31 Lobachevsky St., Kazan, 420111, Russian Federation; b Institute for Cellular and Intracellular Symbiosis, Ural Branch of Russian Academy of Sciences, 11 Pionerskaya St., Orenburg, 460000, Russian Federation; c Institute of Fundamental Medicine and Biology, Kazan (Volga Region) Federal University, 18 Kremlyovskaya St., Kazan, 420111, Russian Federation; d Research Institute of Eye Diseases, 11 Rossolimo St., Moscow, 119021, Russian Federation

**Keywords:** *Salmonella enterica*, Starvation, Low-density population, Rna-seq, Illumina

## Abstract

*Salmonella enterica* is an ubiquitous pathogen throughout the world causing gastroenteritis in humans and animals. Survival of pathogenic bacteria in the external environment may be associated with the ability to overcome the stress caused by starvation. The bacterial response to starvation is well understood in laboratory cultures with a sufficiently high cell density. However, bacterial populations often have a small size when facing this challenge in natural biotopes. The aim of this work was to find out if there are differences in the transcriptomes of *S. enterica* depending on the factor of cell density during starvation. Here we present transcriptome data of *Salmonella enterica* subsp*. enterica* serovar Typhimurium str. 14028S grown in carbon rich or carbon deficient medium with high or low cell density. These data will help identify genes involved in adaptation of low-density bacterial populations to starvation conditions.

Specifications Table

**Table:** 

Subject	Biochemistry, Genetics and Molecular Biology: Molecular Biology
Specific subject area	Transcriptomics
Type of data	Charts, tables, transcriptome sequences
How data were acquired	High-throughput RNA-sequencing
Data format	Raw reads filtered and analysed with statistical tests, FASTQ
Parameters for data collection	Total RNA was extracted from *Salmonella enterica* subsp. *enterica* serovar Typhimurium str. 14028S cells cultured under carbon and phosphorus starvation
Description of data collection	RNA from control and starving samples subjected to RNA-sequencing and transcriptome profiling with subsequent analysis
Data source location	Kazan Scientific Centre of RAS, Kazan, Russia.
Data accessibility	Cleaned FASTQ files are deposited in a public repository: Repository name: NCBI Sequence Read Archive (SRA) Data identification number: PRJNA554270 Direct URL to data: https://www.ncbi.nlm.nih.gov/bioproject/?term=PRJNA554270

## Value of the data

1. For the first time, transcriptome data were obtained for salmonella starved for carbon and phosphorus at high or low cell density.
2. The transcriptome dataset can be used to identify *S. enterica* genes differentially expressed under starvation conditions at high or low bacterial population density.
3. These data can help elucidate mechanisms of persistence used by the pathogenic microorganisms for survival and growth in the oligotrophic biotopes.

## 1 Data description

**Fig. 1:**
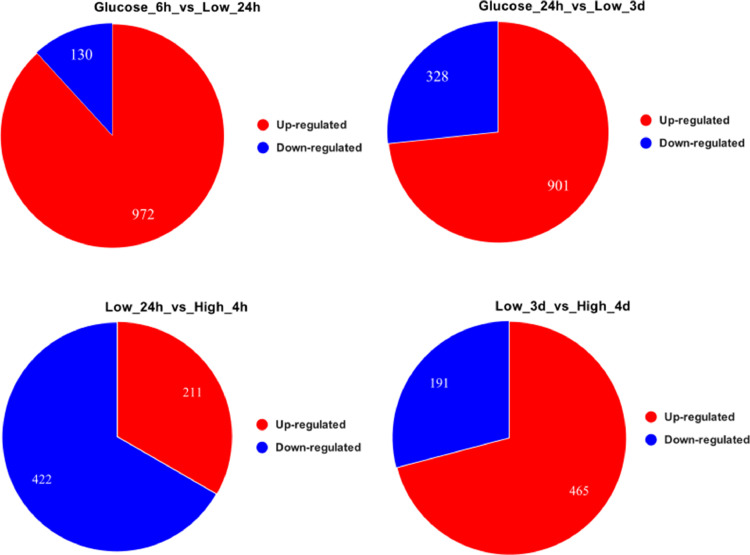
*Salmonella enterica* serovar Typhimurium 14028S transcriptome responses to starvation at high or low cell density. The pie charts show the number of genes with increased or decreased expression at two different time points corresponded to the periods of maximum growth or transition to the stationary phase, respectively.

**Fig. 2:**
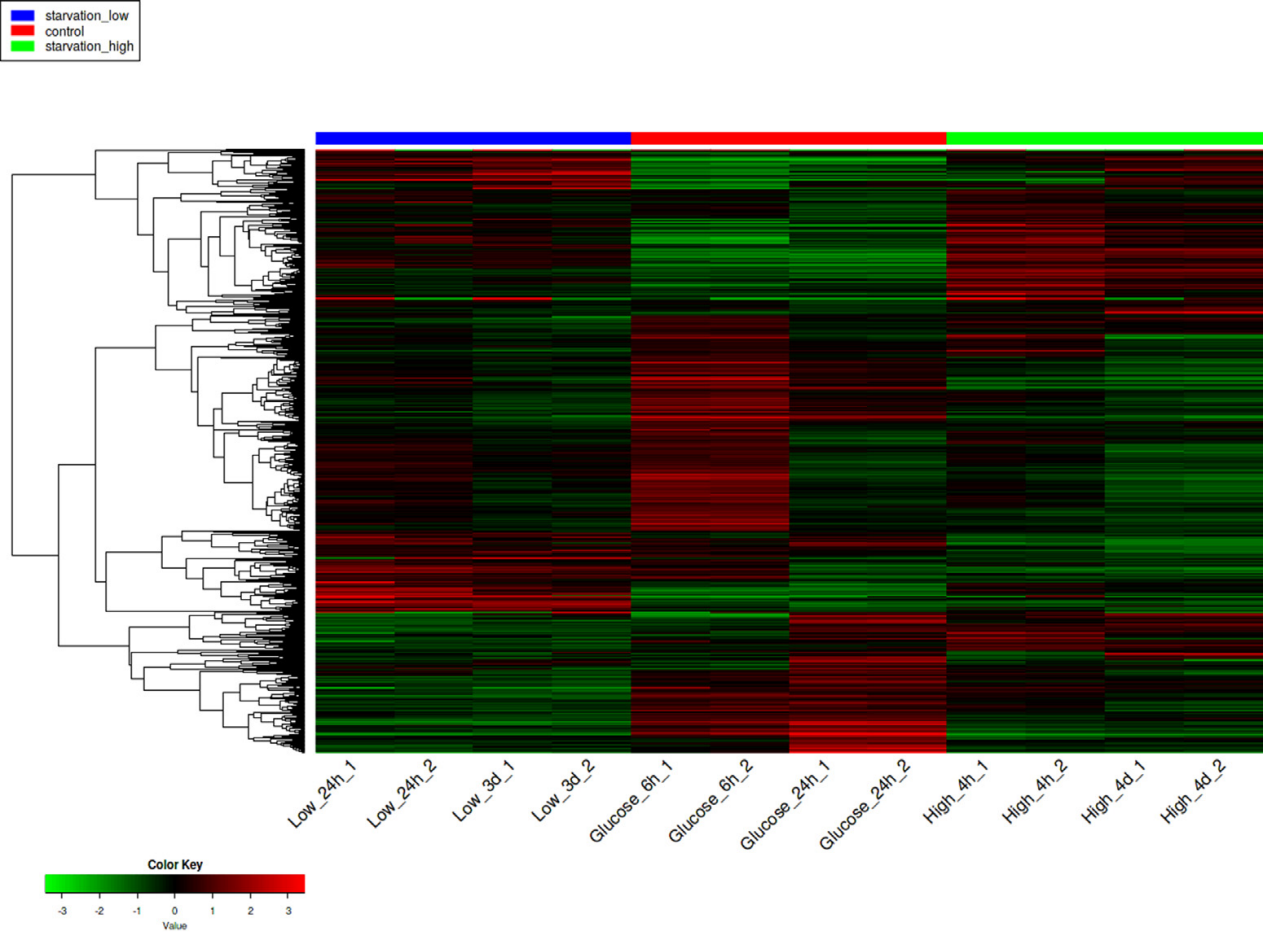
Heat map analysis of 1000 of the most variable genes associated with *Salmonella enterica* serovar Typhimurium 14028S transcriptome responses to starvation at high or low cell density. Hierarchical clustering was constructed based on Pearson correlation distance.

The dataset of this article provides information on raw RNA-seq reads obtained from samples of *Salmonella enterica* serovar Typhimurium 14028S cultures grown in a mineral medium providing carbon and phosphorus starvation, or in the medium supplemented with glucose as a carbon source. The final content of the basic elements in the mineral medium (excluding carbon and nitrogen) is contained in Table 1. Information on the sampling time and growth rate of the cultures is presented in Table 2. This table also provides the NCBI SRA accession numbers of the cleaned FASTQ files for all biological replicates. The transcriptome data obtained are summarized in Table 3. To identify coding and non-coding transcripts, they were mapped on the reference genome. Up- and down-regulated genes were counted with the Differentially Expressed Genes (DEGs) analysis of transcriptomes of the salmonella cultures starved at high or low cell density, as well as the cultures grown in the glucose rich medium (Fig. 1). Besides, we evaluated the identity of 1000 the most variable genes associated with salmonella transcriptome responses to starvation with the heat map analysis (Fig. 2).

**Table 1:** Content of the basic elements in the AB mineral medium.

Element	Concentration, mg/L
K	92.00
S	86.90
Mg	72.10
Na	6.96
Ca	2.40
Fe	0.21
P	0.10
Zn	0.04
Mn	0.01

**Table 2:** Characteristics of *Salmonella enterica* serovar Typhimurium 14028S cultures taken for transcriptome analysis.

Biological replicates	Carbon source	Inoculation titer, CFU/ml	Duration of cultivation, hours / phase of growth or stationary state	Titer of sampled culture, CFU/ml	NCBI SRA accession number
High_4h_1	absent	1 × 10^9^	4 growth	8.0 × 10^9^	SRX7476254
High_4h_2	absent	1 × 10^9^	4 growth	8.1 × 10^9^	SRX7476255
High_4d_1	absent	1 × 10^9^	96 / stationary	5.1 × 10^6^	SRX7476246
High_4d_2	absent	1 × 10^9^	96 / stationary	6.8 × 10^6^	SRX7476247
Low_24h_1	absent	1 × 10^3^	24 / growth	2.2 × 10^5^	SRX7476244
Low_24h_2	absent	1 × 10^3^	24 / growth	2.6 × 10^5^	SRX7476245
Low_3d_1	absent	1 × 10^3^	72 / stationary	4.3 × 10^5^	SRX7476248
Low_3d_2	absent	1 × 10^3^	72 / stationary	6.1 × 10^5^	SRX7476249
Glucose_6h_1	glucose	1 × 10^3^	6 / growth	2.3 × 10^8^	SRX7476250
Glucose_6h_2	glucose	1 × 10^3^	6 / growth	4.9 × 10^8^	SRX7476251
Glucose_24h_1	glucose	1 × 10^3^	24 / stationary	2.0 × 10^8^	SRX7476252
Glucose_24h_2	glucose	1 × 10^3^	24 / stationary	3.1 × 10^8^	SRX7476253

**Table 3:** Number of cleaned reads and reads mapped on the reference genome.

Library	Number of cleaned reads	Number of reads mapped on genome	% Mapped reads	Number of reads mapped on coding sequences	% Reads mapped on coding sequences	% Reads mapped on intergenic regions
High_4h_1	18,406,709	17,908,185	97.3	9475,008	51.48	48.52
High_4h_2	24,679,633	24,254,947	98.3	13,297,127	53.88	46.12
High_4d_1	10,203,859	9865,524	96.7	2477,269	24.28	75.72
High_4d_2	9545,957	9257,803	97.0	2144,698	22.47	77.53
Low_24h_1	20,478,595	19,684,154	96.1	14,737,915	71.97	28.03
Low_24h_2	10,285,237	9705,585	94.4	6413,217	62.35	37.35
Low_3d_1	26,260,086	25,040,464	95.4	8464,941	32.24	67.76
Low_3d_2	8004,395	7588,638	94.8	2107,150	26.32	73.68
Glucose_6h_1	12,440,848	12,193,924	98.6	10,810,167	87.38	12.62
Glucose_6h_ 2	12,826,518	12,443,566	97.6	11,354,492	89.03	10.97
Glucose_24h_1	9851,028	9510,553	96.5	5662,677	57.48	42.52
Glucose_24h_ 2	12,072,222	11,594,716	96.0	7208,000	59.71	40.29

## 2 Experimental design, materials and methods

### 2.1 Strains and growth conditions

In this work *Salmonella enterica* subsp. enterica serovar Typhimurium (ATCC 14,028) strain was used. This strain was cultured on a Luria-Bertani (LB) agar plate (Sigma Aldrich) and a single colony was used to inoculate 10 mL of LB broth. After 12 h aerobic incubation at 37 °C the salmonella cells were collected by centrifugation, washed twice, and transferred to the mineral carbon and phosphorus deficient AB medium containing 1.0 g/L NH_4_Cl; 0.62 g/L MgSO_4_ × 7H_2_O; 0.15 g/L KCl; 0.013 g/L CaCl_2_ × 2H_2_O; 0.005 g/L FeSO_4_ × 7H_2_O, pH – 5.5. Previously, this medium has been used to study the starving cultures of a phytopathogenic bacterium *Pectobacterium atrosepticum* [1,2]. Similar nutrient limited conditions may be found in some natural water bodies and water courses. The elemental composition of the prepared AB medium was checked using an inductively coupled plasma mass spectrometer Aurora M90 (Bruker Corporation, USA). Only a small amount of phosphorus and sodium as additional components was recorded (Table 1). The cell suspensions were incubated under starvation conditions with initial population density of 10^3^ or 10^9^ CFU per ml in glass vials without aeration at 28 °C for 4–96 h before sampling (Table 2). The low-density cultures were also incubated in AB medium supplemented with 1% glucose.

### 2.2 Experiment design

In order to study the dynamics of transcriptome response during salmonella adaptation to starvation, sampling was conducted at two time points corresponded to the periods of maximum growth and transition to the stationary phase, respectively.

In our experiments the number of CFU increased after inoculation in all salmonella cultures. In starving high-density cultures, an eight-fold increase in CFU number observed over four hours was replaced by a gradual decrease, which stabilized by the fourth day of incubation at a final value of about 0.06% of the inoculation titer. Such dynamics of the salmonella culture is in a good agreement with the “altruistic” model of the bacterial stress response associated with programmed death [3]. Based on this dynamics of CFU, the starving high-density cultures were sampled after 4 h and 4 days of incubation (“High_4h” and “High_4d”, Table 2).

Low-density cultures entered the stationary phase just after the period of exponential growth was over. The control low-density cultures in carbon-supplemented AB medium (“Glucose_” in Table 2) were sampled after 6 and 24 h of incubation that corresponded to periods of the maximum growth and the transition to the stationary phase, respectively. However, the growth of experimental low-density cultures incubated in the carbon-deficient medium was significantly impaired. Thus, sampling of these cultures delayed up to 24 h and 3 days (“Low_24h” and “Low_3d”, Table 2).

Total RNA was isolated and cDNA libraries were prepared for RNA-sequencing. Directional libraries were sequenced on Illumina Hiseq 2500 in single reads. The RNA-seq raw reads were stored in FASTQ files, and further analyzed to get the clean reads.

### 2.3 Library construction and sequencing

Bacterial cells were harvested by filtration through nitrocellulose membranes with diameter of pores 0.22 µm (Millipore, USA). The membranes were placed into tubes containing 1 ml of Extract reagent (Evrogen, Russia) and heated at 55 °C 10 min. Then total RNA was extracted according to the manufacturer's protocol. DNA contaminants were removed using RNase-free DNase I kit (Ambion, USA). RNA integrity was checked with an Agilent 2100 bioanalyzer (Agilent,USA). rRNA depletion was performed using Ribo-Zero rRNA Removal Kit for Gram-Negative Bacteria (Illumina, USA). NEBNext Ultra Directional RNA Library Prep Kit for Illumina was used to prepare RNA-seq libraries. The resulting average size of the cDNA libraries was approximately 300 bp. The libraries were sequenced in a single lane of a flow cell on the HiSeq 2500 (Illumina) platform.

### 2.4 Sequence QC and filtering

A total 173,505,388 raw single-end reads 60 bp long were obtained (Table 3). Quality of the raw reads was controlled with FastQC software v. 0.11.3 [4]. Reads belonged to rRNA were removed using SortMeRNA [5]. Trimming of reads and adapters removal were performed with Trimmomatic v.0.35 [6].

### 2.5 Reads alignment to the reference genome and data analysis

Filtered high-quality reads were mapped onto the reference genome sequence of *Salmonella enterica* subsp. *enterica* serovar Typhimurium 14028S assembly GCA_000022165.1 [7] with TopHat 2 [8]. Before mapping the reference genome was indexed with Bowtie2 [9]. Coverage estimates and statistics of the reads mapping are presented in Table 3. Gene level count tables were obtained using the prepDE.py Python script supplemented to Stringtie tool [10]. Differential expression of genes was calculated via DESeq2 [11]. DEGs analysis was carried out for those genes with fold change (FC) 4.0 and fold discovery rate (FDR) adjusted p-value threshold of 0.05

## Author's contribution and ethics statement

**Natalia E. Gogoleva:** Conceptualization, Investigation, Methodology, Validation, Writing - Original draft preparation, Funding acquisition. **Vladimir Ya. Kataev**: Investigation, Writing - Original draft preparation. **Alexander S. Balkin:** Software, Formal analysis, Data curation. **Andrey O. Plotnikov:** Writing - Original draft preparation, Review & Editing. **Elena I. Shagimardanova:** Resources. **Anastasia M. Subbot:** Investigation. **Sergey V. Cherkasov:** Supervision. **Yuri V. Gogolev:** Conceptualization, Methodology, Writing - Original draft preparation, Review & Editing, Funding acquisition.

All ethical requirements were observed in the preparation of the publication. The work was not related to the use of human objects and did not include experiments with animals.

## Declaration of Competing Interest

The authors declare that they have no known competing financial interests or personal relationships that could have appeared to influence the work reported in this paper.

## Appendix Supplementary materials

Image, application 1
